# Supplementary material for: In silico analysis suggests less effective MHC-II presentation of SARS-CoV-2 RBM peptides: Implication for neutralizing antibody responses
Source: PLoS One. 2021 Feb 11;16(2):e0246731. doi: 10.1371/journal.pone.0246731 (PMC7877779; doi:10.1371/journal.pone.0246731)
Supplement: S1 Fig — (PDF) [file pone.0246731.s003.pdf]

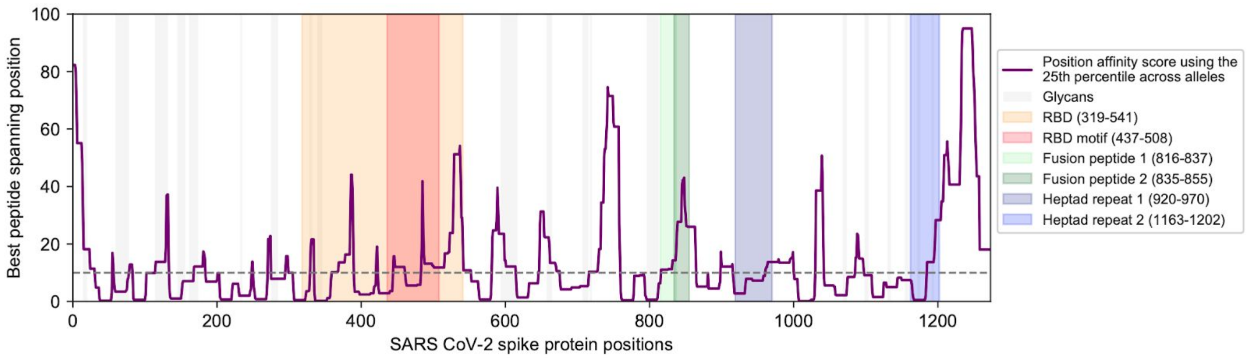

Supplemental Figure 1. Distribution of position scores along the spike protein using the 25th percentile affinity instead of the median affinity.
