# Supplementary material for: In silico analysis suggests less effective MHC-II presentation of SARS-CoV-2 RBM peptides: Implication for neutralizing antibody responses
Source: PLoS One. 2021 Feb 11;16(2):e0246731. doi: 10.1371/journal.pone.0246731 (PMC7877779; doi:10.1371/journal.pone.0246731)
Supplement: S2 Fig — (A) Pileup of corresponding query peptides’ start positions of BLAST-identified peptides that bind to at least one common MHC-II allele. The below barplot shows Fig 3A for reference: the aggregated position scores across supertypes for positions proximal to FNCY. (B) Scatterplot showing the median supertype affinities of BLAST-identified peptides that may bind (median affinity <20) along the corresponding start positions of queried peptides along the spike protein. The FNCY motif region is highlighted in grey. (C) Clustermap showing the median supertype affinities of BLAST-identified peptides that may bind (median affinity <20) to at least one supertype. Median affinities greater than 20 have been adjusted to 20 for better visualization of binding peptides. (PDF) [file pone.0246731.s004.pdf]

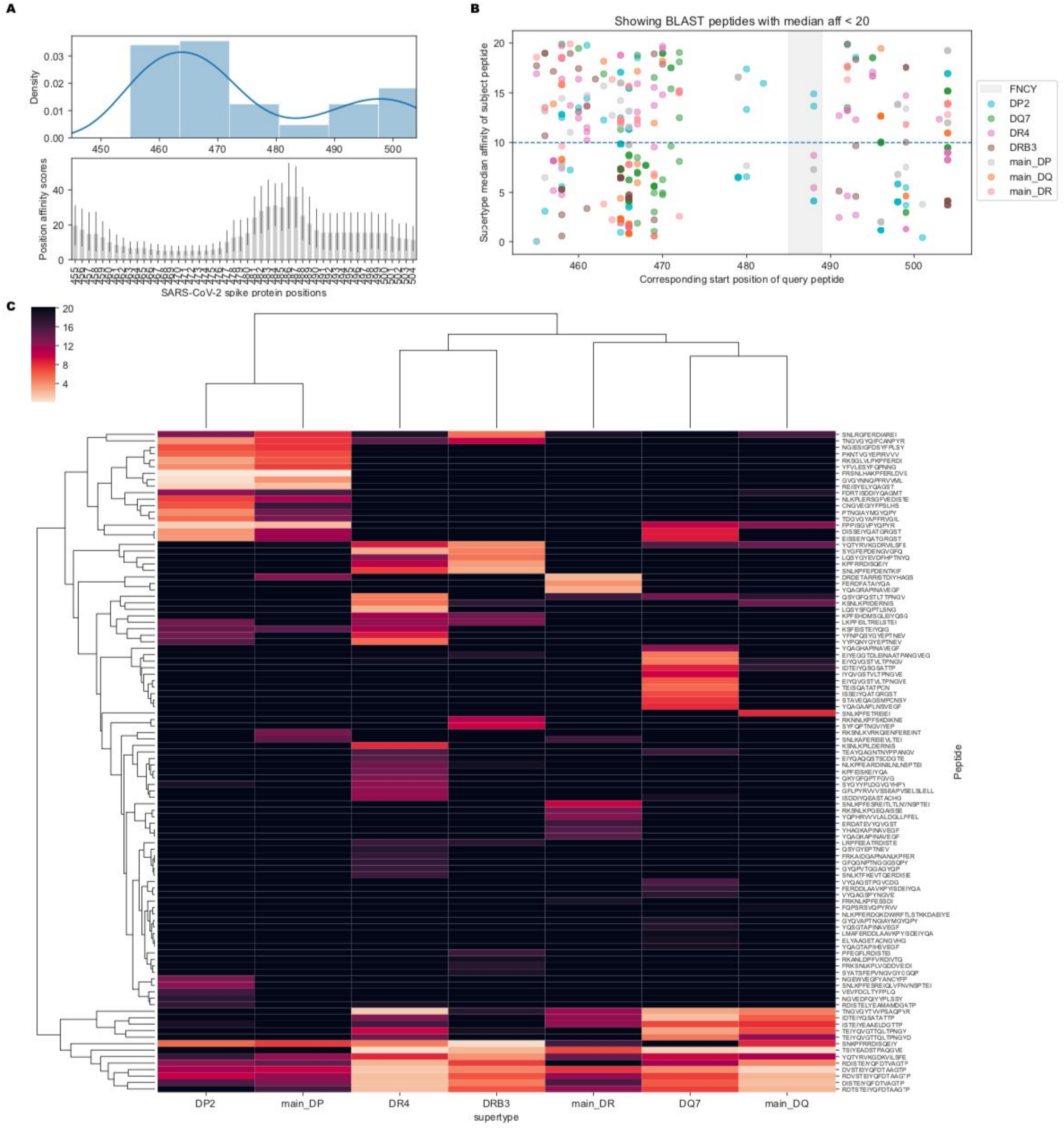

Supplemental Figure 2. Overview of subject peptides that bind at least one retrieved from BLAST search. (A) Pileup of corresponding query peptides' start positions of BLAST-identified peptides that bind to at least one common MHC-II allele. The below barplot shows Figure 3A for reference: the aggregated position scores across supertypes for positions proximal to FNCY. (B) Scatterplot showing the median supertype affinities of BLAST-identified peptides that may bind (median affinity <20) along the corresponding start positions of queried peptides along the spike protein. The FNCY motif region is highlighted in grey. (C) Clustermap showing the median supertype affinities of BLAST-identified peptides that may bind (median affinity <20) to at least one supertype. Median affinities greater than 20 have been adjusted to 20 for better visualization of binding peptides.
