# Supplementary figures and images for: In silico analysis suggests less effective MHC-II presentation of SARS-CoV-2 RBM peptides: Implication for neutralizing antibody responses
Source: PLoS One. 2021 Feb 11;16(2):e0246731. doi: 10.1371/journal.pone.0246731 (PMC7877779; doi:10.1371/journal.pone.0246731)

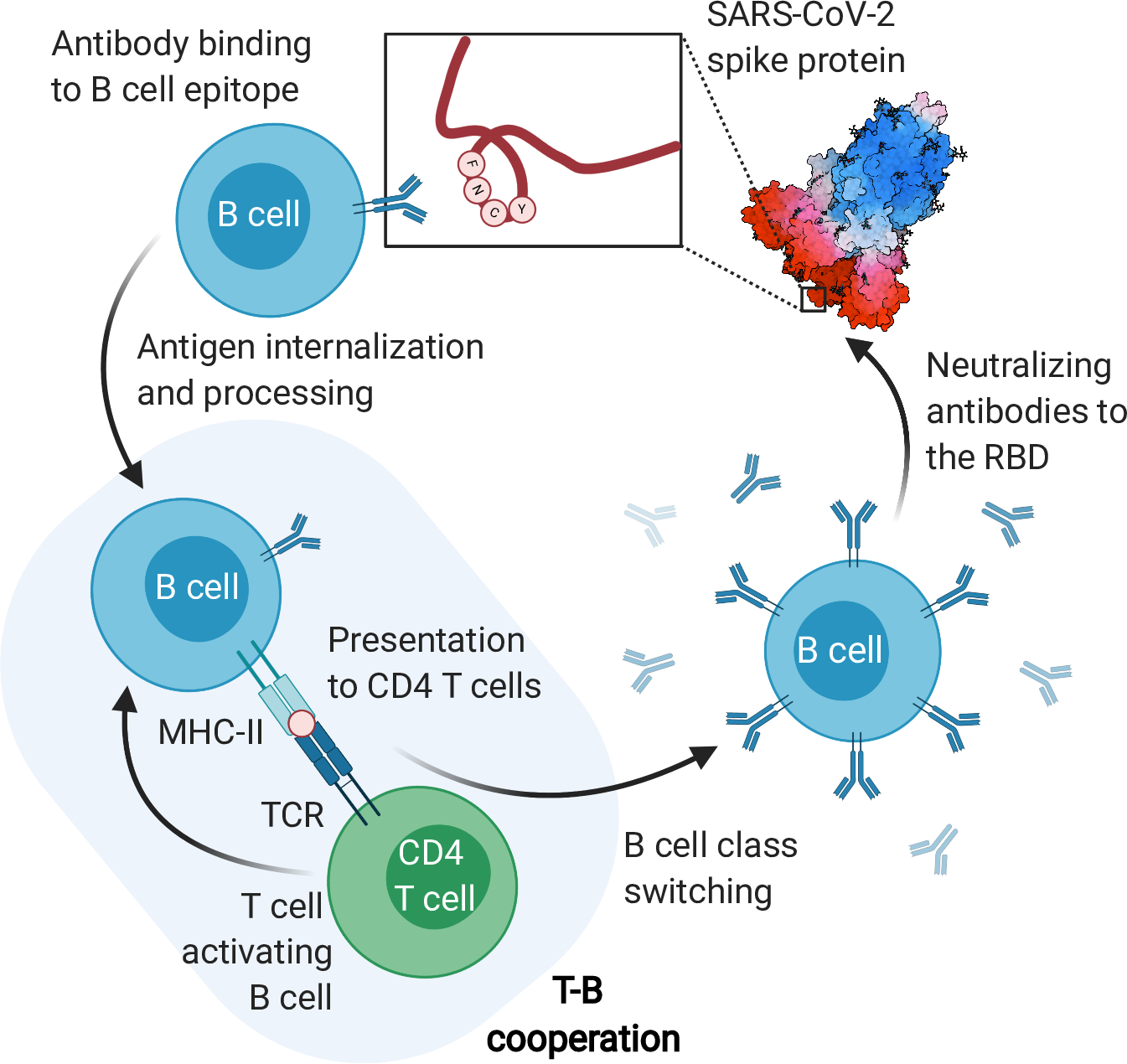

Supplement: S1 Graphical abstract — (TIF) [file pone.0246731.s005.tif]
